# Prevalence, Risk Factors, and Disease Progression of Severe Pneumonia in a Tertiary Hospital in Ethiopia

**DOI:** 10.4314/ejhs.v35i1.5S

**Published:** 2025-12

**Authors:** Samuel Mesfin Girma, Rahel Argaw, Ageru Zeleke, Ayalew Tizazu, Rahwa Amha, Elham Yimam, Fathia Salah, Samuel Sisay, Yimtubezinash Woldeamanuel, Betseat Woldegebriel, Tihitena Negussie Mommo

**Affiliations:** 1 School of Medicine, College of Health Sciences, Addis Ababa University, Ethiopia; 2 Department of Pediatrics, College of Health Sciences, Addis Ababa University, Ethiopia; 3 Department of Surgery, College of Health Sciences, Addis Ababa University, Ethiopia; 4 Institute of Public Health, College of Medical Sciences, University of Gondar; 5 Department of Radiology, College of Health Sciences, Addis Ababa University, Ethiopia; 6 Department of Microbiology, College of Health Sciences, Addis Ababa University, Ethiopia; 7 Department of Pediatrics, Yekatit 12 Hospital Medical College, Ethiopia

**Keywords:** Severe Pneumonia, Disease Progression, Parapneumonic Effusion, Children, Ethiopia

## Abstract

**Background:**

Pneumonia remains the leading cause of mortality and morbidity among pediatric age groups worldwide. Several risk factors contribute to the progression of pneumonia into severe and complicated forms. Parapneumonic effusion is one of the most common complications to consider when severe pneumonia advances to a more critical stage. This study evaluates the prevalence, risk factors, and disease progression of severe pneumonia in children, providing valuable insights for resource-limited settings.

**Methods:**

A hospital-based prospective cohort study was conducted at a tertiary center from July 2022 to December 2023 among children aged 2 months to 14 years with severe pneumonia. Data were collected on socio-demographic characteristics, clinical status, disease progression, microbiological findings, and patient outcomes, and were analyzed using Stata Version 14.

**Results:**

A total of 74.13% of severe pneumonia cases occurred in children under 5 years of age. Fever, cough, tachypnea, dyspnea, and chest indrawing were the most frequently observed clinical presentations. Overall, 31.46% of patients progressed to parapneumonic effusion. Absence of prior pneumonia (AOR = 0.25; 95% CI: 0.11–0.57), no prior hospitalization for the current diagnosis (AOR = 0.49; 95% CI: 0.28–0.86), no exposure to secondhand cigarette smoke (AOR 0.24; 95% CI: 0.07–0.84), and no contact with a coughing patient (AOR = 0.53; 95% CI: 0.29–0.96) were each associated with lower odds of progression to parapneumonic effusion. In contrast, malnutrition was associated with higher odds of progression (AOR = 2.00; 95% CI: 1.18–3.54).

**Conclusion:**

A higher prevalence of severe pneumonia was observed in children under 5 years of age. Disease progression to parapneumonic effusion occurred in 31.46% of cases, and the mortality rate was 4.54%.

## Introduction

Respiratory diseases, including pneumonia, are a leading cause of mortality and morbidity worldwide, with the pediatric population being particularly vulnerable (1). Pneumonia is an acute lower respiratory tract infection that primarily affects the lung parenchyma, leading to alveolar space destruction and impaired gas exchange (2–4). The World Health Organization (WHO) defines pneumonia in children as the presence of cough or difficulty breathing accompanied by rapid breathing or chest indrawing (5). Severe pneumonia is characterized by additional signs such as an inability to drink, persistent vomiting, convulsions, lethargy, stridor, or severe malnutrition (5). Various guidelines outline criteria used to assess pneumonia severity, which often depend on factors such as microbial cause, the potential for targeted or supportive treatment, the likelihood of benefit from experimental therapies, and the risk of morbidity or mortality (6–8). Current evidence highlights hypoxemia, altered mental status (AMS), age below 3–6 months, dyspnea, multilobar infiltrates, and moderate-to-large pleural effusions as key indicators of severe pneumonia in children (9).

Pneumonia remains a significant global health problem affecting over 1,400 children per 100,000 annually, translating to one case per 71 children each year (10,11). The burden is highest in Asia (2,500 cases per 100,000 children) and Africa (1,620 cases per 100,000 children); in 2015, an estimated 138 million episodes of clinical pneumonia occurred in young children, and 32% of countries—predominantly in sub-Saharan Africa and South Asia—were not on track to achieve Sustainable Development Goal (SDG) 3.2 by 2030 (12,13). In 2015, 1.575 million deaths globally were attributed to pneumonia, with approximately 921,000 of these occurring in children under five years of age (14,15). Sub-Saharan Africa experienced high mortality rates, with around 172 deaths per 1,000 live births (16–19). Notably, Ethiopia, along with India, Nigeria, Pakistan, and the Democratic Republic of the Congo, accounted for nearly half (49%) of all pneumonia-related deaths worldwide that year (12). The prevalence of pneumonia among children under five in Ethiopia has been reported to reach as high as 20.68% (20).

Pneumonia progression from mild to severe is a we 11-documented clinical occurrence (21,22). When severe pneumonia advances to a more complicated state, parapneumonic effusion is one of the most common complications to consider, affecting up to 57% of bacterial pneumonia cases (23,24). It occurs when the inflammatory response to lung infection extends across the visceral pleura into the pleural space, leading to fluid accumulation and contributing to increased morbidity and mortality (25).

Risk factors well established for the development of severe pneumonia include younger age, male gender, low weight-for-height, larger family size, low monthly income, use of biomass fuel, malnutrition, lack of immunization, and exposure to cigarette smoke (26–28). Similarly, risk factors for the development of complicated pneumonia include immunodeficiencies, malnutrition, chronic lung diseases, congenital thoracic malformations, inhaled foreign bodies, cardiac diseases, elevated CRP levels, and low oxygen saturation (29,30). Streptococcus pneumoniae is the most frequently identified bacterium in pleural infections, detected in 17.2% of children through blood and pleural fluid samples (31–34).

This study aims to evaluate the risk factors, prevalence, and disease progression of severe pneumonia in children aged 2 months to 14 years admitted to a tertiary hospital within a low- and middle-income country (LMIC). Additionally, it investigates the clinical characteristics and outcomes of children with severe pneumonia, including mortality rates. By identifying key risk factors and disease patterns, this study seeks to provide insights that can inform targeted interventions, optimize treatment protocols, and improve outcomes in resource-limited settings.

## Methods

**Study setting**: Ethiopia, the second most populous country in Africa, is home to over 122 million people (35,36). Tikur Anbessa Specialized Hospital (TASH), a major tertiary care facility affiliated with Addis Ababa University, serves as a critical referral center for the nation. It plays an essential role in pediatric care, attracting patients from across the country. This multidisciplinary study was conducted at TASH from July 2022 to December 2023 (until the target sample size was achieved), incorporating expertise from the Departments of Pediatrics and Child Health, Pediatric Surgery, Radiology, and Microbiology.

**Study design**: A hospital-based prospective cohort study design was employed.

Study population: Participants included children aged 2 months to 14 years admitted to TASH with a diagnosis of severe pneumonia. The age range was selected because the hospital exclusively provides care for children under 14 years of age. Infants younger than 2 months were excluded due to their distinct risk factors—including birth-related complications and immature immune systems—which could influence study outcomes.

**Sample size determination and data collection**: The sample size was calculated using the single-population proportion formula (n = Z^2^P(1–P)/d^2^). Assuming a 35% prevalence of complicated pneumonia, a 95% confidence level (Z = 1.96), and a 5% margin of error, the initial sample size was 350. After applying a finite population correction (<1000), the adjusted sample size was 260. Allowing for a 10% non-response rate, the final sample size was 286.

Data collection proceeded following informed consent from parents/caregivers and assent from patients aged 12–14. A comprehensive questionnaire captured socio-demographic characteristics, risk factors, comorbidities, clinical findings, microbiological analyses, and patient outcomes. Vaccination status was assessed objectively from vaccination cards, either reviewed at the time of data collection or extracted from patient charts.

### Operational definitions

Definitions follow Pediatric Hospital Care: Ethiopia – Guidelines for the Management of Common Illnesses in Hospitals (Second Edition, 2016) and WHO recommendations (37,38).

**Pneumonia**: Cough or difficulty breathing associated with fast breathing or chest indrawing.

**Severe pneumonia**: Pneumonia plus inability to drink, persistent vomiting, convulsions, lethargy, stridor, or severe malnutrition.

**Parapneumonic effusion**: Fluid within the pleural space in a child with pneumonia or severe pneumonia, evidenced by clinical signs such as dullness to percussion, reduced or absent breath sounds, or a pleural rub in early stages, with confirmation on chest radiography.

**Laboratory methods**: Blood cultures were performed using BacT/Alert and incubated for 5 days at 37°C. A culture was considered positive if growth was detected and confirmed by an automated identification system. For coagulase-negative staphylococci, at least two positive cultures drawn separately were required for classification as a true pathogen. C-reactive protein (CRP) levels were measured using the hs-CRP assay on Roche analyzers. A CRP level >10 mg/L was defined as a “marked elevation” (39). Interlaboratory variability was assessed externally three times per year (One World Accuracy), and all laboratories followed CLSI standardized procedures to ensure comparability (40).

**Data analysis**: Data collected using the questionnaire were analyzed with Stata Version 14. Frequency distributions were generated, and bivariate and multivariable logistic regression analyses were conducted to assess potential risk factors for parapneumonic effusion. Variables with p < 0.2 in bivariate analysis were included in the multivariable model.

**Ethical approval**: The study was approved by the Institutional Review Board (IRB) of Addis Ababa University.

**Role of the funding source**: The funding source had no role in study design, data collection, analysis, interpretation, or manuscript preparation.

## Results

The study involved 286 participants, with a male-to-female ratio of 1.18:1. Half of the participants were aged 2–18 months, 24% were aged 18–60 months, and 25.9% were aged 5–13 years. Rural residents constituted 23.4% of the total population (Table 1).

**Table 1:** Summary of socio-demographic characteristics of the study participants

Variable	Category	Frequency	Percent
**Sex**	Male	154	54.0%
	Female	132	46.0%
**Age**	2-18 months	143	50.0%
	18–60 months	69	24.1%
	5-13 years	74	25.9%
**Address**	Addis Ababa	149	52.1%
	Outside of Addis Ababa (Urban)	70	24.5%
	Outside of Addis Ababa (Rural)	67	23.4%

Regarding cooking practices, 45.5% cooked in the main house, while 54.5% used separate kitchens or cooked outdoors. Traditional cooking methods were predominant, with 82.5% using wood, charcoal, or crop residues. In-house cigarette smoke exposure was reported by only 4.55%. Additionally, 6.6% had contact with a known TB patient, and 51.7% had underlying medical or surgical conditions (Table 2). The most common comorbidity was congenital heart disease (CHD), present in 36.8% of participants, followed by malignancy (10.5%), tuberculosis (3.5%), asthma (3.5%), HIV (1.5%), and cerebral palsy (4.6%). Some participants had more than one comorbidity. Pneumococcal vaccination uptake was 100%.

**Table 2:** Summary of risk factors and comorbidities identified among the study participants

Variable	Category		Frequency	Percent
**Cooking Place**	In the main house		130	45.5%
	Separate kitchen		120	42.0%
	Outdoors		36	12.6%
**Cooking Fuel used**	Traditional	Wood	99	34.6%
		Crop residue	8	2.8%
		Improved cooking stove	102	35.7%
		Charcoal	27	9.4%
	Electricity		50	17.5%
**In-house smoking exposure**	Yes		13	4.6%
	No		273	95.5%
**hospitalized before presenting in this hospital**	Yes		158	55.2%
	No		128	44.8%
**Contact with a TB patient**	Yes		19	6.6%
	No		267	93.4%
**Contact with a coughing patient in the past month**	Yes		78	27.3%
	No		208	72.7%
**Underlying medical or surgical conditions**	Yes		148	51.7%
	No		138	48.3%
**Malnutrition**	Yes		140	48.9%
	No		146	51.1%

**Figure 1:**
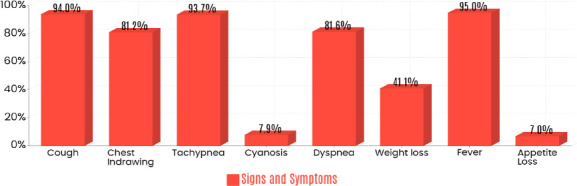
Clinical presentation of the study participants

The majority of participants presented with fever (95%) and cough (94%), the most frequently reported symptoms. Less common symptoms included central cyanosis (7.9%) and appetite loss (7%) (Figure 1). The average symptom duration before admission was 18 days. Only 11.9% reported a history of pneumonia.

**Figure 2:**
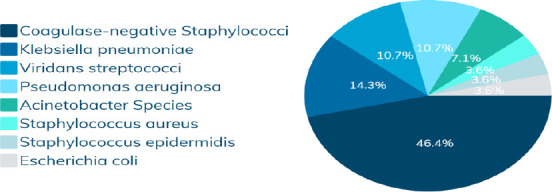
Commonest incriminated pathogens identified from blood cultures of the study participants

Vital sign abnormalities included a mean heart rate of 138.48 ± 20.23 beats/min, respiratory rate of 48.84 ± 16 breaths/min, temperature of 37.66 ± 2.27°C, and oxygen saturation of 84.05 ± 14.01% (with 52% having saturation <90%). Blood work showed an average WBC count of 13.01k ± 12.9k, neutrophils of 56.8 ± 21.7%, and a mean CRP of 34 mg/L, with 57% showing marked elevation. Blood cultures were performed on 58 patients who did not respond to first-line therapy; 29 were positive. Coagulase-negative staphylococci were most common (46.4%), followed by Klebsiella pneumoniae (14.3%), and Viridans streptococci and Pseudomonas aeruginosa (each 10.7%) (Figure 2).

First-line therapy consisted mainly of ceftriaxone-based regimens (81.88%), followed by ampicillin-based (10.14%) and vancomycin-based regimens (5.43%) (Table 3). Second-line therapy was required in 48.6% of participants, guided by blood culture results in 34.5%. Meropenem-based regimens were most common (31.72%), followed by vancomycin-based regimens (28.28%). Clindamycin was the least used (2.76%). Oxygen support was required in 82.52% of participants for a mean of 9.34 ± 8.52 days, and 10.14% required ICU admission.

**Table 3:** Antibiotic regimens used for the treatment of severe pneumonia among the study participants

First-line antibiotic regimen	Prevalence	Second-line antibiotic regimen	Prevalence
Ceftriaxone-based	81.88%	Meropenem-based	31.72%
Ampicillin-based	10.14%	Vancomycin-based	28.28%
Vancomycin-based	5.43%	Augmentin	15.86%
Azithromycin-based	1.09%	Cefepime-based	14.48%
Meropenem-based	1.45%	Ceftriaxone-based	6.90%
		Clindamycin	2.76%

Approximately 31.46% progressed to parapneumonic effusion, with 53.3% of affected individuals being male and a mean age of 4.48 ± 3.64 years. Variables with p < 0.2 in bivariate analysis were included in multivariable regression. VIF values ranged from 1.2–2.8, indicating no significant multicollinearity (41). Sensitivity analyses showed stable adjusted odds ratios.

In multivariable analysis, the following were significantly associated with parapneumonic effusion (Table 4): no previous pneumonia history (AOR = 0.25; 95% CI: 0.11–0.57), prior hospitalization for the current diagnosis (AOR = 0.49; 95% CI: 0.28–0.86), no in-house cigarette smoke exposure (AOR = 0.24; 95% CI: 0.07–0.84), no contact with a coughing patient in the past month (AOR = 0.53; 95% CI: 0.29–0.96) and malnutrition (AOR = 2.00; 95% CI: 1.18–3.54).

**Table 4:** Bivariate and Multivariable logistic regression analysis results

Variable	Category	Effusion		COR	P-value	AOR	P value

		No	Yes				
**Age**	2-18 months	98	35	-	-		
	18-60 months	42	21	0.79(0.42-1.51)	0.48		
	5-13 years	56	34	1.3(0.76-2.46)	0.29		
**Address**	Addis Ababa	104	45	-	-	-	-
	Urban outside of Addis Ababa	48	22	1.05(0.57-1.95)	0.854		
	Rural	44	23	1.20(0.65-2.23)	0.546		
**Cooking place**	Main House	89	41	-	-	-	-
	Separate Kitchen	76	44	1.25(0.74-2.12)	0.393	1.5(0.86-2.74)	0.145
	Outdoors	31	5	0.35(0.12-0.96)	0.043**	0.41(0.14-1.2)	0.107
**Cooking fuel**	Wood	65	34	-	-		
	Crop Residue	4	4	1.91(0.44-8.12)	0.38		
	Improved cooking stove	75	27	0.68(0.37-1.25)	0.22		
	Charcoal	20	7	0.66(0.25-1.73)	0.41		
	Electricity	32	18	1.07(0.52-2.18)	0.84		
**Previous**	Yes	13	21	-	-	-	-
**Pneumonia**	No	183	69	0.23(0.11-0.49)	0.00**	0.25(0.11-0.57)	0.001*
**Hospitalized before the current admission**	Yes	97	61	-	-	-	-
	No	99	29	0.46(0.27-0.78)	0.004**	0.49(0.28-0.86)	0.014*
**In-house cigarette smoke exposure**	Yes	5	8	-	-	-	-
	No	191	82	0.26(0.08-0.84)	0.025**	0.24(0.07-0.84)	0.026*
**Contact with a coughing patient**	Yes	46	32	-	-	-	-
	No	150	58	0.55(0.32-0.95)	0.034*	0.53(0.29-0.96)	0.039*
**Malnutrition**	Yes	83	57	2.35(1.40-3.93)	0.001**	2(1.18-3.54)	0.011*
	No	113	33	-	-	-	-

Note:

(**) candidate for multivariable logistic regression at p-value < 0.2

(*) statistically significant at p-value < 0.05

The average hospital stay was 12 ± 9.75 days. The mortality rate was 4.54%, with all deaths attributed to respiratory failure secondary to septic shock.

## Discussion

This study examined the prevalence, risk factors, and progression of severe pneumonia among children at Ethiopia's largest tertiary hospital. The most commonly affected age group was 2–18 months, representing about 50% of cases; children under 5 accounted for 74.13%, consistent with previous studies (18,42). Males were slightly more affected, aligning with earlier findings (43).

Most participants presented with fever (95%), cough (94%), tachypnea (93.7%), dyspnea (81.6%), and chest indrawing (81.2%), findings consistent with previous studies (4,44,45). Vitalsign abnormalities—including tachycardia, tachypnea, fever, and hypoxia—were also consistent with published data (46,47).

Laboratory results revealed leukocytosis with neutrophilia and elevated CRP, expected in severe pneumonia (45–47). Blood cultures identified coagulase-negative staphylococci as the most common pathogen, followed by Klebsiella pneumoniae, Viridans streptococci, and Pseudomonas aeruginosa. This contrasts with earlier studies highlighting Streptococcus pneumoniae, Staphylococcus aureus, and Haemophilus influenzae as predominant pathogens (51,52). The differences may reflect both environmental variations and the fact that cultures in this study were performed only in patients not responding to first-line therapy, potentially selecting for more resistant or atypical organisms.

Most participants received ceftriaxone- or ampicillin-based first-line therapy. Nearly half required second-line antibiotics, often meropenem-or vancomycin-based, typically after lack of improvement over 17 ± 13.7 days. Over 80% required oxygen support. ICU admission occurred in 10.14%—lower than previous reports of 13.8–14.5% (53,54).

Parapneumonic effusion occurred in 31.5% of participants—lower than the 57% reported in some studies (23,24). Significant risk factors identified were in-house cigarette smoke exposure, malnutrition, previous pneumonia, prior hospitalization for pneumonia, and recent contact with a coughing person—consistent with earlier findings (26–30). Average hospital stay was 12 ± 9.75 days. Mortality was 4.54%, lower than previously reported rates of 11.4–12.3% (55,56). All deaths resulted from respiratory failure secondary to septic shock, consistent with earlier studies (57).

In conclusion, this study highlights a high prevalence (74.13%) of severe pneumonia among children under five. Fever, cough, tachypnea, dyspnea, and chest indrawing were the most common clinical features, with leukocytosis, neutrophilia, and elevated CRP as frequent laboratory findings. Parapneumonic effusion developed in 31.46% of severe pneumonia cases and was associated with in-house cigarette smoke exposure, malnutrition, prior pneumonia, prior hospitalization, and recent contact with a coughing person. Most cases were successfully treated with first-line antibiotics. The mortality rate was 4.54%.

The findings underscore the importance of effective antibiotic therapy, addressing modifiable risk factors, and developing targeted treatment strategies to improve outcomes for pediatric patients with severe pneumonia. Future research should incorporate time-to-recovery survival analyses to evaluate treatment options and explore additional interventions using a more representative sample size.

This study has several limitations. Blood cultures were performed only in patients who failed first-line therapy, which may have biased results toward resistant or atypical pathogens and underestimated overall pathogen diversity. Although aseptic technique was used, repeat cultures and confirmatory diagnostics (e.g., PCR, pleural fluid cultures) were not performed, increasing the possibility of contamination—particularly with organisms such as coagulase-negative staphylococci. Additionally, as the study was conducted in a tertiary referral hospital that predominantly manages severe and complicated cases, the findings may not reflect the broader population. This referral bias may have led to an overestimation of certain risk factors, such as malnutrition and prior hospitalization, while the relatively low mortality rate likely reflects access to advanced supportive care. Thus, the findings are most applicable to tertiary-care settings.
